# Ethical, methodological, and contextual challenges in research in conflict settings: the case of Syrian refugee children in Lebanon

**DOI:** 10.1186/s13031-019-0215-z

**Published:** 2019-06-25

**Authors:** Rima R. Habib

**Affiliations:** Department of Environmental Health Faculty of Health Sciences, American University of Beirut, P.O.Box: 11-0236 Riad El Solh, Beirut, 1107 2020 Lebanon

**Keywords:** Research, Conflict setting, Fragility, Syrian refugees, Ethics, Challenges, International development

## Abstract

Research within conflict settings challenges the ethical assumptions of traditional research practice. The tensions between theory and practice were evident in a study of working children among Syrian refugee communities in Lebanon. While the study sought to introduce scientific evidence that might support effective policy solutions, its implementation was marked by a struggle to navigate bureaucracy, vested political interests, climates of xenophobia and sectarianism, and an unfolding military conflict that cast a shadow on the research initiative.

The study pushed the researcher to examine privileged understandings of research ethics and elucidated structural, institutional, and societal obstacles beleaguering efforts to support refugees. Many of the challenges of the research process were structural in nature, tethered to the institutional and societal contexts within which the research was conceived and conducted. Some of these entrenched dynamics may be inescapable within the parameters of institutional research, while others may be addressed through greater awareness and preparation. Specifically, researchers studying refugee communities within conflict settings must intentionally reflect on the dynamics that govern refugee politics in the research context. Particular attention must be paid to the elements of xenophobia, violence, and fear that impact participants’ autonomy and agency within the study.

Intentional engagement with these dynamics cannot insulate the research process from the coercive realities of the refugee experience, yet researchers do have the opportunity to transparently reaffirm their commitments to ethical practice.

## Background

Research within conflict settings challenges the ethical assumptions of traditional research practice. While most bioethicists maintain that the ethics guiding research during peacetime are the same as those during war [1], this expectation is unrealistic with the study of refugee populations near conflict zones. To study refugee populations is to participate in a political struggle being waged by powerful interests who are attempting to shape the narratives that dictate public policies and perceptions. This analysis presents reflections on the complicated experiences of implementing a study of child labor among Syrian refugees in Lebanon. This vulnerable subset of refugees face daily challenges to their security, wellbeing, and survival [2]. The research study sought to make visible the realities of working children and introduce scientific evidence that might support effective policy solutions. These experiences revealed that engaging in this discourse was inherently controversial, implicating powerful geopolitical interests and arousing xenophobic backlash that undermined the integrity of the research process. The study pushed the researcher to examine privileged understandings of research ethics and elucidated structural, institutional, and societal obstacles beleaguering efforts to support refugees.

### Syrians refugees in Lebanon

The recent war in Syria (2011 and ongoing) resulted in a crisis of displacement and forced migration of catastrophic proportions. Syrians now constitute the largest group of refugees worldwide, with an estimated 3,606,737 fleeing to Turkey, 938,531 to Lebanon, and 660,393 to Jordan [3]. A small country, Lebanon has disproportionately felt the impact of this crisis and currently has the highest number of refugees per capita globally [4]. Life in Lebanon for most Syrian refugees is difficult: 75% of Syrian refugee households have no access to basic food, shelter, health and education and 58% live in extreme poverty [5]. Refugee poverty is due in part to a poor national economy, the absence of national legal frameworks protecting refugee rights, and inadequate international aid resources to address the scope of the humanitarian crisis [6–8].

The refugee population has also suffered from inconsistent governmental policies. Since the onset of the war in Syria and the resulting refugee influx, the Lebanese government has taken numerous measures to regulate the entry, stay, and work of Syrian refugees. Among these measures, the government has halted registration with the United Nations High Commission on Refugees (UNHCR) and imposed harsh residency requirements and employment restrictions [8–10]. These policies have effectively pushed child refugees into the labor force, as children face fewer restrictions on their movement [11]. Syrian children working as street beggars, vendors, and farm workers have become instrumental to the survival of many Syrian families residing in Lebanon [12, 13].

The refugee crisis has dramatically impacted the Lebanese economic, social, and political landscape. In 2015, primary school enrolment reached 113.5% [14] and national unemployment has doubled since 2011 [15]. Housing availability has also become increasingly scarce, leading to rent rises and overcrowding. The evident impacts of the crisis have generated resentment among Lebanese nationals, who feel that refugees pose a threat to national sovereignty [16]. This populist nationalism has been fueled by the presence of ISIS and Nusra militants among the refugee population and the sectarian violence that has followed.

### Obstacles to urgency within research

In late 2015, the Child Labor Unit at the Lebanese Ministry of Labor in coordination with the International Labour Organization (ILO), the United Nations International Children’s Emergency Fund (UNICEF) and the Food and Agriculture Organization of the United Nations (FAO) approached the Faculty of Health and Sciences (FHS) at the American University of Beirut (AUB) to undertake an assessment of child labour among Syrian refugees in the agricultural sector of Lebanon’s Bekaa Valley. A research team from AUB prepared a study of Syrian refugee communities located near agricultural areas in the Bekaa, a region with the largest refugee population in the country [17]. From its outset, the research was beset by logistical challenges.

**Fig. 1:**
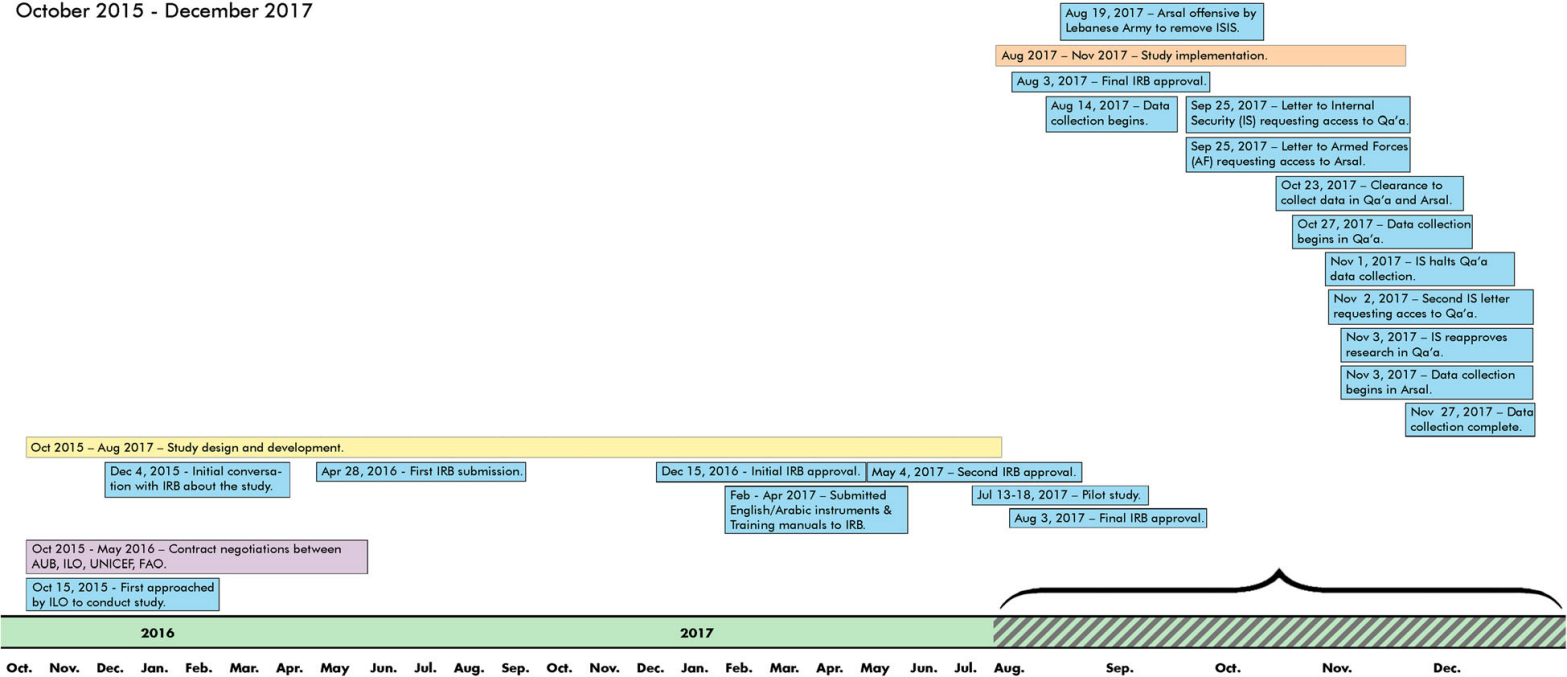
Study Timeline

A crisis of this magnitude presented an urgent mandate to provide policymakers with evidence to inform their decision-making. Despite the stated urgency of project partners, the initiative progressed slowly as the research team navigated massive bureaucracies, a lengthy Institutional Review Board (IRB) process, and delays to study implementation due to ongoing military activities. Figure 1 illustrates the study chronology.

The lengthiest aspects of the research process were the contractual negotiations (7 months) and IRB approval (17 months), compared to a 4 month implementation phase (see Study Timeline in Fig. 1). These extended timeframes were the result of a research process that engaged multiple large bureaucratic entities, each with deliberate and litigious approaches to decision making. AUB and the UN organizations negotiated over organizational roles and sub-contractor bidding. Complicating matters, each UN organization had independent contract negotiations, stating stipulations that sometimes conflicted with those of other partnering organizations.

Other political considerations guided the study design. Each agency wished to include research components that were guided by their own priorities, which had practical impacts on the logistics of the study. There were also negotiations over the study’s scale that reflected these organizations’ perceptions of the aims of the research. Specifically, the organizations’ original intent was for a small-scale study that could quickly validate established UN policy agendas. They felt that a quantitative study would be more convincing to policymakers. The research team noted that a small-scale quantitative study would not produce meaningful findings, suggesting a larger quantitative study instead. This proposal had drawbacks in that it required more time and funding than the UN organizations originally provisioned. Aware of these parameters, the UN organizations agreed to the proposed study and to AUB researchers seeking funds from other organizations. However, this decision created pressures on the research team in meeting the requirements of the different funding entities.

As anticipated, the project was delayed by a lengthy IRB process. The IRB was particularly exacting on the study’s application because of the “multiple vulnerabilities” of the study population. The IRB raised concerns about consent, employer retaliation, and other potential consequences facing the child refugees. Due to these factors, the project protocol required 20 months to receive IRB approval starting with initial meetings with IRB staff to discuss the application requirements, introducing modifications to the study protocol, lodging the IRB application on 28 April 2016, responding to several cycles of revision, including a final revision of the protocol following the pilot study. The project received final IRB approval on 3 August 2017. The main issues raised by IRB in the subsequent revisions included (1) the wording and sensitivity of the questions in the research instruments and the consent and assent forms, (2) the controversy over the relevant sections of the Lebanese Laws that govern the protection of refugees and the reporting of abuse among refugee child workers, (3) the process of follow-up on cases of abuse reported by the child participants. A consequence of this deliberate IRB process was the elapse of deadlines and contracts with UN agencies and local aid organizations. Despite a thorough IRB process, there were still gaps in addressing the ethical challenges facing the study.

These experiences demonstrate the obstacles to timely scholarship within conflict settings. In the 26 months required to complete the study, the refugee crisis dramatically changed. The research team anticipated such delays, informing the UN organization in 2015 that research conducted through an academic institution would be slow moving. The decision to commit to this approach seemingly conflicted with the situational urgency. It is possible that the UN organizations were pressured by other stakeholders to develop a study through AUB. The decision-making process guiding this research was evidently beholden to various organizations, interests, and agendas. These political considerations are inherent to research during crisis and should be a forthright consideration of researchers engaging in this field.

### Xenophobia under conflict

The global resurgence of populist nationalism and the consequent criminalization of migrants [18] has reshaped the landscape of refugee research. Faced with the baseless assumptions that undergird xenophobic ideation, scientific inquiry can serve as an important tool to counter hate [19]. Through intentional agenda setting, study design, and analysis, scholars can actively counter ideologically-fueled misinformation and perspectives. Evidently, refugee research is often viewed as transgressive by stakeholders whose authority and interests are implicated by this work. Understanding how xenophobia affects research necessitates nuanced analyses of context and power.

In North America and Europe, ethno-nationalists mobilize xenophobic tropes that criminalize migrants and refugees to advance nationalist and racist political agendas [18]. Similarly, some Lebanese politicians utilize xenophobic discourse for political gain, yet their rhetoric derives from a unique context [20]. Unlike Western countries where fears of societal collapse are overblown, every facet of daily life in Lebanon has been impacted by the crisis and many Lebanese have legitimate fears that a prolonged crisis poses an existential threat to the nation. The impact of the refugee crisis on the Lebanese psyche has fueled resentment towards both Syrian refugees and an international community which has simultaneously enflamed the Syrian conflict while withholding resources to address the refugee crisis.

With these dynamics at play, Lebanese xenophobia could be framed in connection with immediate economic and security concerns. A prominent example was the mass-eviction of refugees near Zahle in the Bekaa Valley. In early 2017, ISIS coordinated a suicide bombing attack against Christian residents in Qa’a, killing eight. This led the local governor to pressure the Lebanese army to evict 300 Muslim refugees from their homes, although there was no evidence that these families were involved in the attacks [21]. This incident, alongside several other highly publicized mass evictions, impacted refugees’ sense of safety across the country and had ramifications for the research process. During data collection, refugees voiced mistrust of research team members. One mother was hesitant to participate fearing eviction if she revealed her family’s violation of child labor laws. Others were anxious that researchers might be gathering intelligence for the armed forces. These interactions highlighted the importance of providing clear assurances to participants that the research team would ensure their anonymity and confidentiality.

The pervasiveness of anti-refugee sentiments were evident throughout the research process. Remarks that the refugees were changing the country and deteriorating the quality of life in Lebanon were often heard during the fieldwork. A Lebanese person in the field was overheard calling Syrian refugees “unclean” and bringing misery upon themselves “by procreating unlimitedly”. These xenophobic statements mirrored similar talking points on television referring to refugee fertility as a “demographic threat” to Lebanon. Anxieties around demographic changes tied into already established sectarian narratives that a large number of Sunni Syrian refugees might shift Lebanon’s sectarian balance of power.

This xenophobic climate likely impacted the study outcomes. One piece of evidence suggesting this impact were the reporting patterns of child deaths on the job. The majority of these incidents were reported to a Syrian team member from the same region as many of the refugees. Participants may have been less trusting of Lebanese members of the field team contributing to underreporting of certain outcomes. Ideally, most members of the research team would have been Syrian refugees; however, the partner organizations insisted on hiring a Lebanese NGO to subcontract the fieldwork, while AUB policy limited the hiring of Syrian workers. The research team may have also been more proactive in anticipating the impact of xenophobia on the study. Future studies operating in contexts with similar tensions might engage team members in intentional discussions about the impact of xenophobia on the research process.

There were other ways in which xenophobia impacted the research process. Societal resentment towards refugees has informed the ways in which Lebanese communities interact with agencies working with refugees, particularly when refugees are perceived as receiving special treatment over underserved locals. Arising out of this dynamic is a “both…and” discourse which demanded that aid agencies provide resources to *both* underserved Lebanese residents *and* Syrian refugees. This approach mirrors the “interest-convergence” framework of Derrick Bell, which argues that support for socially marginalized populations will only receive critical acceptance when aligned with the interests of dominant groups [22]. The research team appealed to interest convergence throughout the research process, for example employing mostly Lebanese nationals as field researchers instead of hiring and training Syrian refugees to lead these activities. Syrian research team members would have likely been more effective for the research, promoting trust and comfort; however, the research team recognized the necessity of accruing goodwill among Lebanese residents. The research team also made intentional efforts to appeal to Lebanese interests while presenting the study findings to officials in the national government. Specifically, we highlighted the relevance of our findings and recommendations to both Syrian and Lebanese nationals.

### Sectarianism, conflict, and the refugee crisis

Sectarian political dynamics inherent to Lebanon have also permeated important aspects of the research initiative, particularly the ways in which the research team related to feelings of safety and security. In mid-2017, there were military skirmishes between the Lebanese government and predominantly Sunni ISIS and Nusra militants hiding in the mountains above Arsal, an area where data collection took place. Many of the militants had family members confined to the informal camps in the valley below, which were encircled by the Lebanese army. Military skirmishes had been commonplace since 2014 in that area, but the conflict intensified following a series of violent escalations by militants. ISIS and Nusra carried out bombings in Dahieh, a predominantly Shi’a suburb of Beirut and in Qa’a, a Christian village in the Bekaa [23, 24]. Further, the militants near Arsal had captured and beheaded several Shi’a Lebanese soldiers stationed in the area, prompting military operations to recover the soldiers’ bodies [25]. The Lebanese Army waged weeks of military operations that successfully led to a ceasefire and withdrawal of militants from the area.

With the cessation of military action, the research team engaged in a month-long negotiation with Internal Security and the Lebanese Armed Forces, eventually receiving permission to resume activities in Qa’a and Arsal. By the time permission was granted, the contract for the NGO conducting field activities had expired. The research team decided to hire new field research staff, connecting with local leaders in Qa’a and Hermel (a village not far from Arsal). The hiring of new team members took on a sectarian dimension because of the segregation that exists in the Lebanese countryside. A gatekeeper in Qa’a explicitly referenced sectarian expectations, insisting that the research team hire local Lebanese Christians to conduct the research in the encampments adjacent to Qa’a. When the research team approached a stakeholder in Hermel, they offered to hire area residents to collect data in Arsal. Although this person did not explicitly reference sectarian hiring preferences, Hermel residents were Shi’a, resulting in a research team with that sectarian identity.

The sectarian identities of the new field team members had a varied impact on the research. The Christian workers from Qa’a perceived their work as “safe” because the camps they were working in were closely surveilled by the Lebanese Internal Security Forces. Arsal, on the other hand, was not perceived as safe by the Hermel workers. The refugees in Arsal predominantly came from Qusayr, a Syrian village where there were bloody fights between Sunni and Shi’a. As Shi’a, the Hermel research team were aware of these histories of sectarian violence and knew that they would be walking into the homes of families whose relatives fought Shi’a based on their religious identity. Despite assurances from the military that the refugees’ homes were safe, members of the research team expressed serious anxieties. Many of the Hermel researchers were visibly Shi’a, wearing chadors and speaking with identifiable accents. These sectarian markers likely contributed to the research team’s fears. However, once the work began, many of the presumptions of sectarian tensions dissipated. The Hermel research team found the refugees gracious and inviting.

Sectarianism was experienced as a dynamic obstacle to the research process. While the observed instances of sectarianism were unique to the context of this conflict and research project, feelings of mistrust towards people from other sects is quite common in Lebanon. Similar to the observations of xenophobia, the research process could not be insulated from a ubiquitous feature of Lebanese society. The experience of the Hermel research team does show an ancillary effect of evidence-based social science research: by facilitating intercommunity interactions and promoting greater understanding, research can promote compassion and diversity.

### Consent under duress

The research establishment considers consent to be a lynchpin of ethical research conduct. Yet scholars who work with vulnerable populations contest the feasibility of consent within contexts where culture and/or circumstances apply coercive pressure for participation [26–29]. Ellis et al. critique informed consent as based in western notions of individual autonomy and self-determination that do not necessarily apply in cultures with a more collective orientation [26]. Consent is an even more precarious possibility for refugees who are frequently subjected to mandatory cooperation and live in environments where they are not free from reprisals or intimidation [30]. Despite the research team’s efforts to protect participants’ autonomy, the coercive realities of refugee life in Lebanon likely impacted study participation. Participation rates in the study were unusually high (97%) compared to what is common in social science research. A multitude of unintended coercive factors likely impacted this participation rate.

One dynamic was the alignment of the research team with powerful institutions and aid organizations that were active in the areas being canvassed. During data collection, research team members informed the refugee participants that the study was a joint effort of AUB, UN organizations, and a local NGO. In presenting this information, participants were assured that their decision to partake would not lead to retaliatory actions. However, the people who were approached may not have been willing to risk the chance that non-participation would impact their already vulnerable standing.

Another factor that complicated informed consent was the use of local gatekeepers for recruitment. The research team approached local *shaweesh*—a sort of employment broker for the refugee communities–to identify tents that housed families with child laborers [31, 32]. The shaweesh were useful in many instances because of their intimate knowledge of the communities living in each ITS. However, reliance on this gatekeeper may have also had a coercive effect on participation rates. Refugee households may have felt compelled to participate knowing that their home was referred to by their employment broker. There may have also been another layer of unintended coercion in the recruitment of the shaweesh to support data collection efforts. The shaweesh themselves may have felt compelled to support the project as the field team informed them of the approval of the Lebanese municipal authority to engage in this research. The shaweesh often operated on land owned by Lebanese municipalities, and so referencing this approval may have forced shaweesh cooperation.

Initially, the study was not going to go through the shaweesh to identify households as the research team wished to implement a completely randomized sampling methodology. However, the pilot study revealed the difficulties of identifying households with working children. The field workers pointed out that refugee households living in the visited ITSs had working children, but household respondents would often deny this fact. For many of the households, revealing that their children are working might have been perceived as a possible reason for deportation, further displacement, or ineligibility for aid services. Other households might not have openly discussed the issue because they felt ashamed that their children must work – particularly in jobs like garbage picking. This obstacle to data collection was navigated with the help of the local shaweesh, who gave assurances that refugee participation was safe.

Another dynamic which impacted participation was the experience of militarization among refugees living in Arsal and Qa’a, who experienced violence in a way that completely compromised their autonomy. Residents of Arsal and Qa’a refugee camps were subject to longstanding military surveillance and presence, with one resident reporting that a family member was beaten by the army because he was suspected of helping ISIS and Nusra. Given these precarious living conditions, members of the research team could not request the participation of community members in a way that precluded the possibility of coercion. Researchers were affiliated with a prestigious American institution and multiple UN organizations and had the blessing of the Lebanese army to operate in the community. Despite vocal efforts to assure residents that there would be no consequences for non-participation, refugees may not have felt free to withdraw their participation.

## Conclusion

The ethical challenges of conducting research with Syrian refugees in Lebanon are defined by power, politics, and necessity. The research team struggled to implement a study while navigating bureaucracy, vested political interests, climates of xenophobia and sectarianism, and an unfolding military conflict that cast a shadow on the research initiative. With the absence of definitive guidelines on conducting refugee research, the research team was forced to improvise and address challenges as they arose in real time. Unfortunately, many of these challenges were structural in nature, tethered to the institutional and societal contexts within which the research was devised and conducted. These entrenched dynamics may be inescapable within the parameters of institutional research. Furthermore, the political nature of refugee research initiatives may undermine good science and proper ethical practice.

That said, the study identified potential interventions to improve the health and wellbeing of refugee working children and their families [17]. It also provided an opportunity to reflect on the ethics, design and implementation of research in contexts of conflict and fragility. Foremost is the need to operate with a focus and intentionality around the complex dynamics that govern refugee politics and discourse in the research context. Researchers must consider how these dynamics might impact the research process from the macro to the micro. Particular attention must be paid to the elements of xenophobia, violence, and fear that impact participants’ sense of agency to participate freely (or not) within the study. The purpose of an intentional engagement with these dynamics is not to solve or insulate the research process from their corrosive effects; rather, these oppressive dynamics are often inescapable elements of the research context and must be considered as potential sources of bias and opportunities for research to reaffirm commitments to ethical practice.

## Data Availability

Not applicable.

## Abbreviations

AUB: American University of Beirut, a prominent English-language research university located in Beirut, Lebanon
FAO: Food and Agriculture Organization of the United Nations is a specialized agency of the United Nations that leads international efforts to defeat hunger. Serving both developed and developing countries, FAO acts as a neutral forum where all nations meet as equals to negotiate arguments and debate policy
FHS: Faculty of Health and Sciences, an academic department at the American University of Beirut
ILO: International Labour Organization is a UN agency promoting decent work for all women and men through a tripartite model involving governments, employers and workers
IRB: Institutional Review Board is a type of committee that applies research ethics by reviewing and approving the methods proposed for research to ensure that they are ethical
ISIS: Islamic State of Iraq and Syria, an extrastate militant organization seeking to establish a global Islamic caliphate through armed conquest
NGO: Non-governmental organization is a nonprofit organization that operates independently of any government, typically one whose purpose is to address a social or political issue
UN: United Nations is an intergovernmental organization that was tasked to maintain international peace and security, develop friendly relations among nations, achieve international co-operation and be a centre for harmonizing the actions of nations
UNHCR: The Office of the United Nations High Commissioner for Refugees is a United Nations programme with the mandate to protect refugees, forcibly displaced communities and stateless people, and assist in their voluntary repatriation, local integration or resettlement to a third country
UNICEF: United Nations International Children’s Emergency Fund helps provide emergency food and healthcare to impoverished children globally
